# Upregulation of the EMT marker vimentin is associated with poor clinical outcome in acute myeloid leukemia

**DOI:** 10.1186/s12967-018-1539-y

**Published:** 2018-06-20

**Authors:** Sharon Wu, Yang Du, John Beckford, Houda Alachkar

**Affiliations:** School of Pharmacy, University of Southern California, Los Angeles, CA USA

**Keywords:** Vimentin, AML, Overall survival, EMT

## Abstract

**Background:**

Vimentin (VIM) is a type III intermediate filament that maintains cell integrity, and is involved in cell migration, motility and adhesion. When overexpressed in solid cancers, vimentin drives epithelial to mesenchymal transition (EMT) and ultimately, metastasis. The effects of its overexpression in AML are unclear.

**Methods:**

In this study, we analyzed the TCGA data of 173 AML patients for which complete clinical and expression data were available. In this analysis, we assessed the association between *VIM* mRNA expression and patient’s clinical and molecular characteristics including clinical outcome.

**Results:**

*VIM* overexpression was associated with higher white blood count (< p = 0.0001). Patients with high *VIM* expression have worse overall survival (OS) and disease-free survival (DFS) compared with patients with low *VIM* expression (median OS; 7.95 months vs 19.2 months; p = 0.029). After age-stratification, high *VIM* expression was significantly associated with worse overall survival in older patients (age ≥ 60; median OS: 5.4 vs 9.9 months: p = 0.0257) but not in younger patients (age < 60). In stratification analysis according to cytogenetic status, high *VIM* expression was significantly associated with shorter OS (7.95 vs 24.6 months: p = 0.0102) in cytogenetically normal, but not in cytogenetic abnormal AML.

**Conclusions:**

Collectively, the data indicate that overexpression of the EMT marker vimentin is associated with poor clinical outcome in older patients with cytogenetically normal AML; and therefore may play a role in this disease.

**Electronic supplementary material:**

The online version of this article (10.1186/s12967-018-1539-y) contains supplementary material, which is available to authorized users.

## Background

Acute myeloid leukemia (AML) is a myeloid lineage malignancy characterized by invasion of the bone marrow and blood by poorly differentiated cells with enhanced proliferative capabilities. Deregulation of various kinases, transcription factors, apoptotic regulators, epigenetic modifiers, etc. and their roles in AML progression have been rigorously studied and are well understood [1]. The deregulation of cytoskeletal proteins particularly those involved in the epithelial to mesenchymal transition (EMT) is well investigated in solid cancers but not in hematological malignancies such as AML. Vimentin (encoded by VIM) a type III intermediate filament, forms the cytoskeletal network with microtubules and microfilaments. It is the predominant intermediate filament in mesenchymal cells and leukocytes [2, 3]. A highly conserved protein, vimentin participates in crucial cellular processes such as cell motility, migration and adhesion as well as promotes resistance to various cell stressors [2, 4, 5]. Furthermore, though vimentin is a cytoskeletal protein, it has been shown to be involved in apoptotic progression [4, 6–8]. During apoptosis, vimentin is cleaved by caspase-3, -7 and -6 resulting in cytoskeletal collapse and is thought to be the basis of the morphological changes that occur in a cell undergoing apoptosis [7]. Direct phosphorylation of vimentin by *AKT1*, a known cancer promoting kinase, protects it from caspase mediated cleavage and blocks apoptotic progression [9, 10]. Given that vimentin regulates cell migration and adhesion, which are crucial processes in cancer progression, it has long been seen as a factor in cancer progression. Vimentin overexpression has been reported in cancers of the prostate, gastrointestinal tract, breast and many others [10–12]. In solid cancers, vimentin has been shown to promote metastatic progression by participating in the cytoskeletal reorganization that occurs during EMT as well as regulate pro-EMT signaling pathways [10]. Vimentin has also been used as a marker for pre-metastatic cells undergoing EMT and therefore, high vimentin expression is associated with worse outcomes in patients with solid cancer [11].

Vimentin is recognized as a player in solid cancer progression but its function in blood cancers, particularly AML, is less clear as leukemic cells do not undergo EMT. We speculate that in AML, vimentin may act as a negative regulator of apoptosis, promote cell motility, confer increased resistance to various stressors as well as regulate signaling networks that promote leukemic cell survival. Based on this speculation, we hypothesize that high *VIM* expression is associated with more aggressive disease and worse outcomes in AML patients.

## Methods

### Patients and treatment

Data on patients with complete clinical and RNA expression data from the cancer genome atlas (TCGA) dataset were included in this study. We identified 173 patients with AML (median age 58 years; range 18–88). These patients were diagnosed and received treatment according to National Comprehensive Cancer Network (NCCN) guidelines between November 2001 and March 2010 [13]. Patients in the intermediate and poor cytogenetic risk groups did not receive uniform treatment: they were included in clinical trials and received allogeneic stem cell transplants whenever applicable or when matched donors were available. In our analysis, we adjusted for transplant status [13]. In total, 91 patients (52.6%) were aged < 60 years and 82 patients (47.4%) were aged 60 ≥  years. The diagnosis of AML as well as risk group stratification were done according to NCCN guidelines. The patients were assigned subtype classifications according to the French–American–British (FAB) classifications. The patients included in the study were assessed for gene expression as well as somatic mutations frequently found in AML, such as *FLT3*, *NPM1*, *IDH1/2*, *TET*, etc. Patient clinical, gene expression (Z score), mutations, gene methylation and survival data were downloaded from the TCGA database on March 10, 2018 via cBioportal [14, 15].

### Gene expression analyses

Publicly available analyzed RNA sequencing data were downloaded from TCGA. Expression values (Z-scores) were used to dichotomize patients into two groups based on *VIM* expression data Z ≥ 1 and Z < 1. In additional analysis we divided patients into Z ≥ 2 and Z < 2.

### Statistical analyses

The time between diagnosis and removal from study due to lack of complete remission, relapse, or death was defined as disease free survival (DFS). The time between diagnosis and death due to any reason was defined as overall survival (OS). Kaplan–Meier survival were generated for the comparison of overall and disease-free survival between patients with Z ≥ 1 and Z < 1 *VIM* expression. In order to determine associations between *VIM* expression levels and patient clinical/molecular characteristics, Mann–Whitney U’s non-parametric and Fisher’s exact test were used for continuous and categorical variables, respectively, using STATA 12.0 SE. Figures were generated using GraphPad Prism software package (ver. 5.0; GraphPad Software Inc., La Jolla, CA, USA). The Stata 12.0 SE software was utilized to perform multivariate analysis using the Cox Proportional Hazards Model to assess the association between *VIM* expression and OS as well as DFS after adjusting for other clinical factors. We also conducted survival analysis by dichotomizing patients according to age (< 60) into younger and older (≥ 60) and excluding patients with t15:17 inversion. A statistical cut-off of p < 0.05 was used for inclusion of variables from univariate analysis to multivariate analysis.

## Results

### *VIM* expression in AML samples

**Fig. 1:**
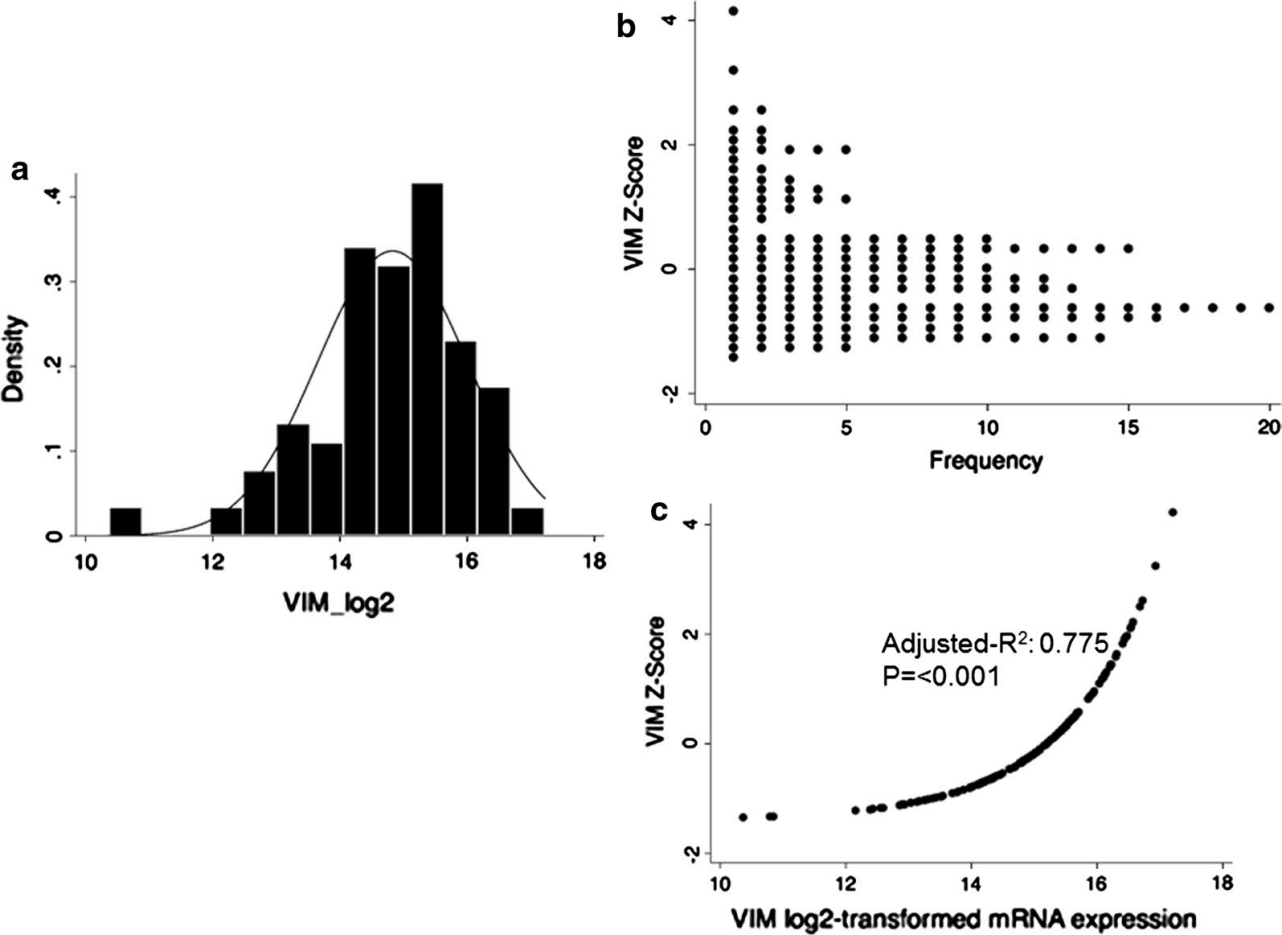
*VIM* Z-score and mRNA expression distribution. **a** Histogram of *VIM* (log2 transformed) mRNA expression; **b** dotplot of *VIM* mRNA expression Z-score; **c** scatterplot of *VIM* log2 mRNA expression vs *VIM* Z-scores

*VIM* mRNA expression (RNA Seq V2 RSEM) data were downloaded from TCGA; and log2 transformed. Histograms depicting the distribution of these mRNA log2-transformed data and *VIM* Z scores are shown in Fig. 1a, b, as well as a scatter plot of *VIM* log2-transformed mRNA expression against *VIM* Z-score (Fig. 1c: adjusted-R^2^: 0.775, p ≤ 0.001). Based on the distribution of *VIM* Z scores, we used a Z score cut off of 1 and 2 for further analysis.

In order to assess *VIM* expression in primitive progenitor cells, we analyzed the AML-GSE30377 data. This set provides gene expression data of cells obtained from 23 patients with AML and sorted into stem cells and progenitors according to CD34 and CD38 markers. *VIM* expression was not found to be higher in the sorted population enriched for the stem cells and the progenitors (CD34+/CD38−) and (CD34+/CD38+) populations. However, we found that *VIM* was significantly lower in the CD34−/CD38− cells compared to unsorted cells (Additional file 1: Figure S1).

### Association between *VIM* expression and patient primary characteristics

**Fig. 2:**
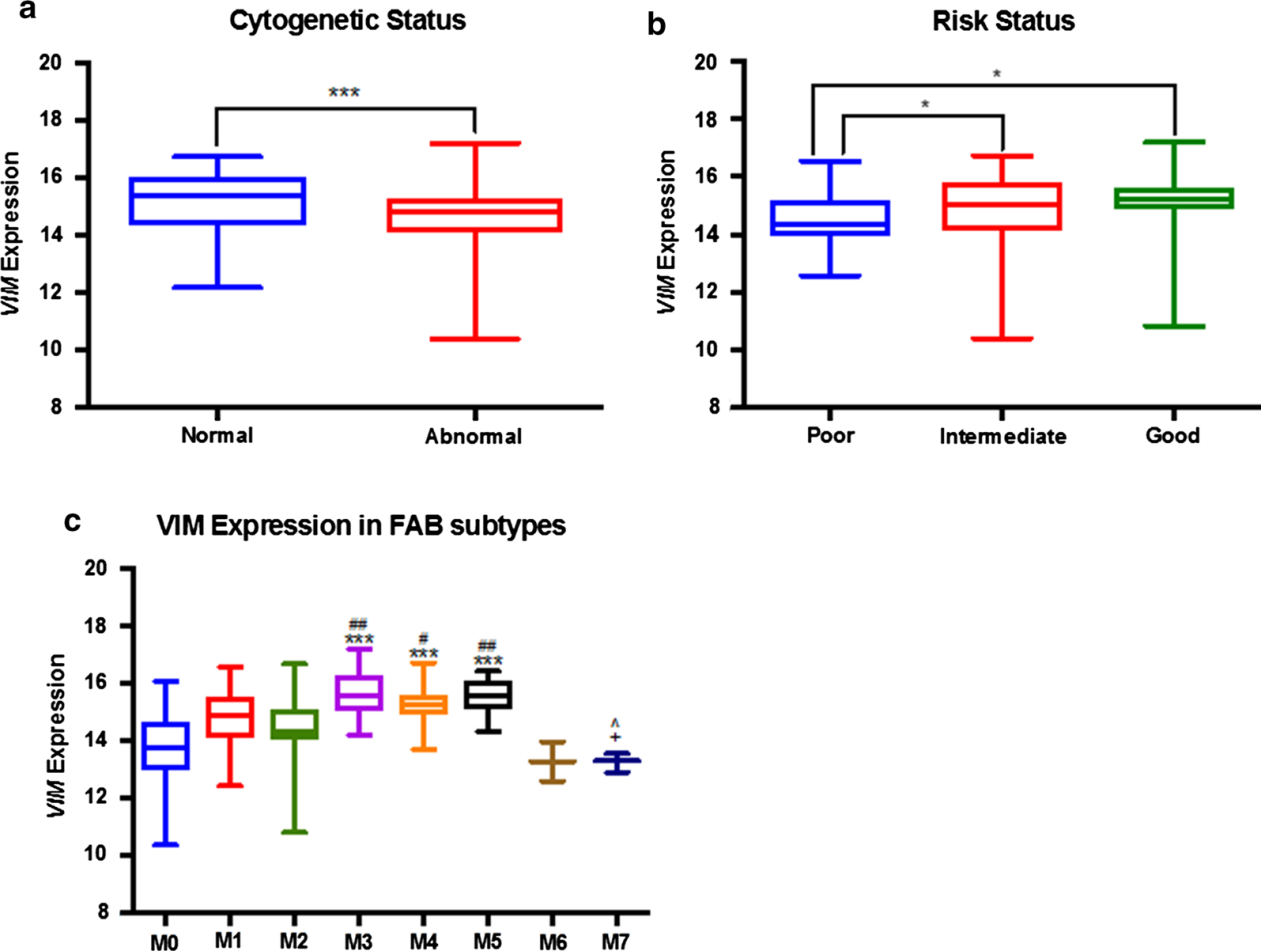
*VIM* mRNA expression in patients with AML according to cytogenetic, risk and FAB classifications. *VIM* log2 mRNA expression categorized by **a** cytogenetic status, **b** risk status and **c** FAB classification: asterisk compared to M0, hash symbol compared to M2, plus sign compared to M3 and cap symbol compared to M5. *p < 0.05; ***p < 0.001

We analyzed *VIM* mRNA expression (log2-transformed) of AML patients based on cytogenetic risk and cytogenetic status. We found that median *VIM* mRNA expression was significantly higher in cytogenetically normal (CN-AML) patients compared to cytogenetically abnormal (CA-AML) patients (Fig. 2a: 15.4 vs 14.8; p = 0.0007). Additionally, median *VIM* mRNA expression was significantly lower in patients with poor cytogenetic risk compared to intermediate and favorable risk (Fig. 2b: p = 0.0011). Median *VIM* mRNA expression was significantly lower in M0 and M2 FAB classes compared to M3, M4 and M5 FAB classes of AML (Fig. 2c: p ≤ 0.0001).

Next we categorized patients according to *VIM* Z score into high (Z ≥ 1) and low (Z < 1) and assessed the association between high *VIM* expression (Z ≥ 1) and patients clinical characteristics (Table 1). We found that 28 patients (16%) have high *VIM* expression (Z ≥ 1). Patients with high *VIM* have higher WBC counts (median: 69.2 vs 12.6, p < 0.001) and higher bone marrow blasts (median %: 82.5 vs 71, p = 0.017) than patients with low *VIM*. High *VIM* expression was also significantly associated with age (median: 63.5 vs 57; p = 0.048), normal cytogenetic (%: 71.4 vs 41.4, p = 0.003) and transplant status (%: 21.4 vs 46.2; p = 0.021). There was no significant association between high *VIM* expression (Z ≥ 1) and FAB subtype, sex, or median peripheral blood blast percentage. However, when we considered patient with Z ≥ 2 for high *VIM* expression, we found that high *VIM* is associated with advanced age (median: 69 vs 57, p = 0.036), median white blood cell count (median: 69.2 vs 15.1; p = 0.016) and increased peripheral blast counts (median %: 65 vs 33, p = 0.022) (Additional file 1: Table S1).

**Table 1:** Clinical characteristics of 173 AML patients according to *VIM* expression Z-score ≥ 1

Characteristic	Z-score (< 1) (n = 145)	Z-score (≥ 1) (n = 28)	p value
Age, median (years)	57	63.5	0.048
Young (< 60 years)	81 (55.9%)	10 (64.3%)	0.063
Old (> 60 years)	64 (44.1%)	18 (35.7%)	
Sex			
Female (n, %)	68 (46.9%)	13 (46.4%)	> 0.999
Male (n, %)	77 (53.1%)	15 (53.6%)	
FAB			
M0 (n, %)	15 (10.3%)	1 (3.57%)	0.476
M1 (n, %)	39 (26.9%)	5 (17.9%)	0.353
M2 (n, %)	34 (23.5%)	4 (14.3%)	0.329
M3 (n, %)	11 (7.59%)	5 (17.9%)	0.146
M4 (n, %)	27 (18.6%)	7 (25.00%)	0.446
M5 (n, %)	12 (8.28%)	6 (21.4%)	0.083
M6 (n, %)	2 (1.38%)	0 (0.00%)	> 0.999
M7 (n, %)	3 (2.07%)	0 (0.00%)	> 0.999
WB count, median	12.6	69.2	< 0.001
ln (WB count), mean	2.446	3.749	< 0.001
% BM blast, median	71	82.5	0.017
% PB blast, median	34	44.5	0.253
Risk status			
Poor (n, %)	42 (29.0%)	3 (10.7%)	0.057
Intermediate (n, %)	73 (50.3%)	19 (67.9%)	0.091
Good (n, %)	28 (19.3%)	5 (17.9%)	> 0.999
Cytogenetic status			
Normal (n, %)	60 (41.4%)	20 (71.4%)	0.003
Abnormal (n, %)	83 (57.2%)	7 (25.0%)	
Transplant status			
No (n, %)	78 (53.8%)	22 (78.6%)	0.021
Yes (n, %)	67 (46.2%)	6 (21.4%)	

### Association between *VIM* expression and patient mutational status

Furthermore, we analyzed *VIM* expression according to patient’s mutational status (Table 2). *VIM* high expression (Z ≥ 1) was found to be associated with *NPM1* mutation (%: 46.4% vs 24.1%; p value: 0.021), but this association was lost when considering Z-score ≥2 (%: 37.5 vs 27.3%; p value: 0.687, Additional file 1: Table S2). No other association was found between *VIM* expression and patient’s mutational status.

**Table 2:** Expression of *VIM* (Z-score ≥ 1) according to the top mutations present in AML (N = 173 patients)

Genes	Z-score (< 1) (n = 145)	Z-score (≥ 1) (n = 28)	p value
FLT3 (n, %)	41 (28.3%)	8 (28.6%)	> 0.999
TP53 (n, %)	14 (9.66%)	0 (0.00%)	0.130
NPM1 (n, %)	35 (24.1%)	13 (46.4%)	0.021
NRAS (n, %)	10 (6.90%)	2 (7.14%)	> 0.999
TET2 (n, %)	14 (9.66%)	1 (3.57%)	0.470
RUNX1 (n, %)	14 (9.66%)	1 (3.57%)	0.470
CEBPA (n, %)	12 (8.28%)	1 (3.57%)	0.696
WT1 (n, %)	10 (6.90%)	0 (0.00%)	0.369
DNMT3A (n, %)	34 (23.4%)	8 (28.6%)	0.631
IDH1 (n, %)	13 (8.97%)	3 (10.7%)	0.726
IDH2 (n, %)	13 (8.97%)	4 (14.3%)	0.484

### Patients with high *VIM* expression have shorter overall and disease-free survival

**Fig. 3:**
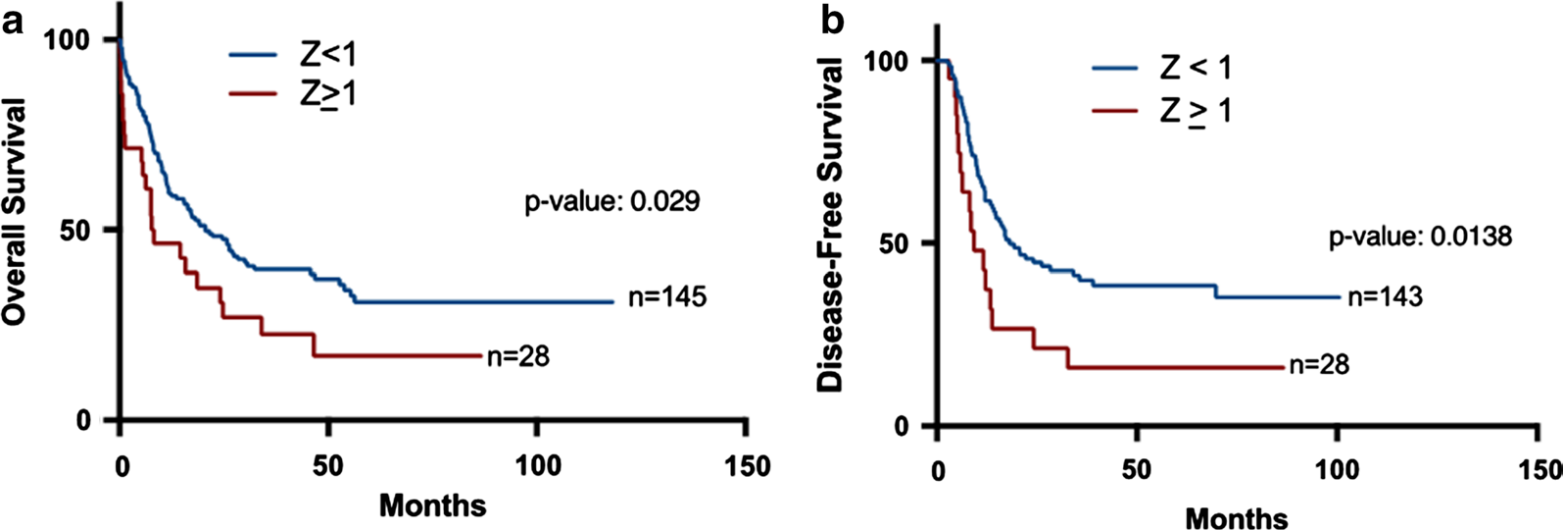
Survival analysis of AML patients associated with *VIM* expression. **a** Overall survival of 173 AML patients with *VIM* Z-score ≥ 1 and *VIM* Z-score < 1; **b** disease-free survival of 171 AML patients with *VIM* Z-score ≥ 1 and *VIM* Z-score < 1

**Fig. 4:**
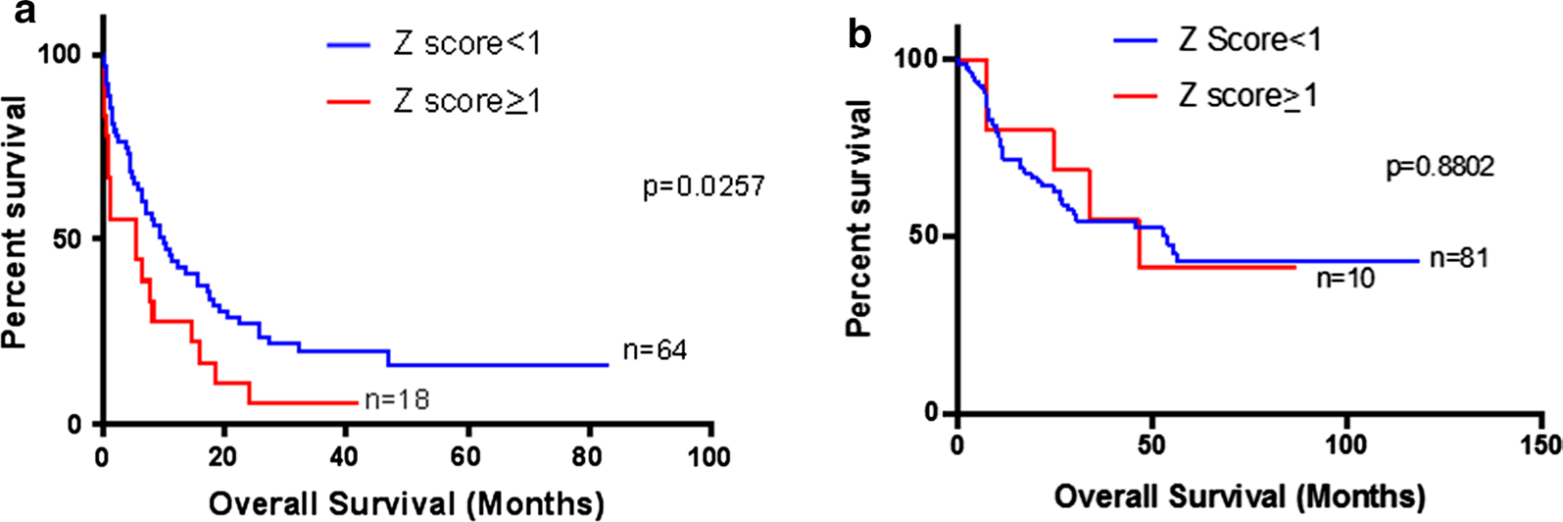
Survival analysis of AML patients associated with *VIM* expression after age-stratification. **a** Overall survival of AML patients with *VIM* Z-score ≥ 1 and *VIM* Z-score < 1 in older patients (age ≥ 60). **b** Overall survival of AML patients with *VIM* Z-score ≥ 1 and *VIM* Z-score < 1 in younger patients (age < 60)

**Fig. 5:**
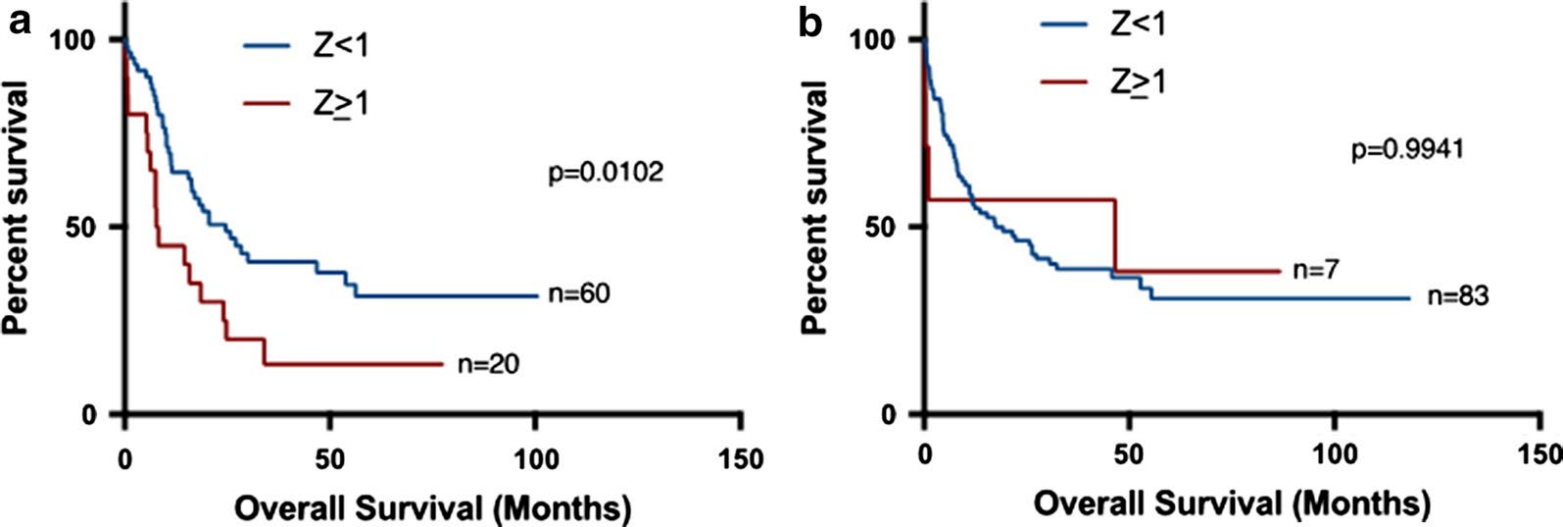
Survival analysis of AML patients associated with *VIM* expression after stratification according to cytogenetic status. **a** Overall survival of AML patients with *VIM* expression *VIM* Z-score ≥ 1 and *VIM* Z-score < 1 in cytogenetically normal patients. **b** Overall survival of AML patients with *VIM* expression *VIM* Z-score ≥ 1 and *VIM* Z-score < 1 in cytogenetically abnormal patients

We compared overall survival and disease-free survival (OS and DFS, respectively) between patients with high (Z ≥ 1) and low (Z < 1) *VIM* expression. We found that patients with high *VIM* expression (Z ≥ 1) had significantly shorter median OS than patients with low *VIM* expression (median: 7.95 months vs 19.2 months; p = 0.029) (Fig. 3a). Similarly, high *VIM* expression patients had significantly shorter DFS compared with low *VIM* expression patients (median: 5.65 months vs 10.8 months; p = 0.0138) (Fig. 3b). After age stratification, high *VIM* expression was still significantly associated with worse overall survival in older patients (Fig. 4a: age ≥ 60—median 5.4 vs 9.9 months: p = 0.0257) but not in younger patients (Fig. 4b: age < 60). Importantly, when we stratified patients according to the cytogenetic status; we found that in CN-AML, but not in CA-AML, high *VIM* expression (Z ≥ 1) was significantly associated with shorter overall survival (Fig. 5a, b: 7.95 vs 24.6 months: p = 0.0102). Stratification of patients according to transplant status showed a similar trend of an association between high *VIM* expression and shorter OS, however it did not reach statistical significance. (no transplant—Additional file 1: Figure S2A: 6.9 vs 10.2 months: p = 0.1607; received transplant—Additional file 1: Figure S2B: 24.8 vs 30.6 months: p = 0.5265).

**Fig. 6:**
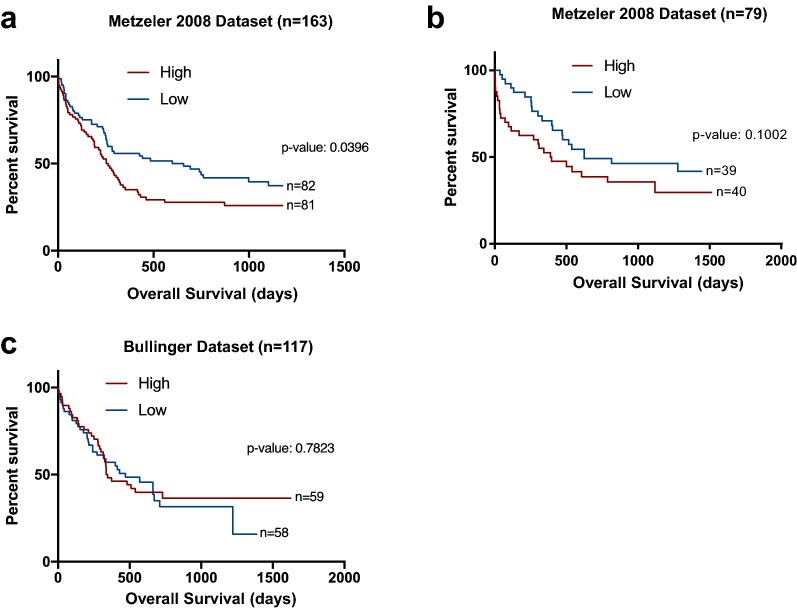
Survival analysis of patients with AML associated with *VIM* expression in three different datasets. Overall survival of patients with AML in various datasets after dichotomization of *VIM* mRNA expression into high and low according to the log-2 median-centered expression: **a**, **b** CN-AML (Metzeler, Blood, 2008) and **c** CN-AML and CA-AML (Bullinger, NEJM, 2004)

We analyzed the association between *VIM* expression and clinical outcomes in three other datasets: two Metzeler and one Bullinger datasets. Because Z scores were not available in these datasets, we dichotomized patients according to median *VIM* expression into high (above median) and low (below median). High *VIM* expression was significantly associated with worse overall survival (Fig. 6a: n = 163: 263 vs 657 days; p value: 0.0396). Similarly, the second Metzeler dataset (Fig. 6b: n = 79: 392 vs 624 days; p value: 0.1002) showed a non-significant but similar trend towards worse overall survival associated with high *VIM* expression, both Metzeler datasets include CN-AML patients only [16]. Contrastingly, the Bullinger (n = 119) dataset which includes only 45 patients with CN-AML, showed no significant association between *VIM* expression and clinical outcome (Fig. 6c) [17].

After adjusting for other risk factors associated with disease free survival, multivariate analyses indicated that high (Z ≥ 1) *VIM* expression was associated with shorter DFS in patients with AML (HR: 2.42 95% CI 1.26–4.66; p = 0.008, Table 3). The association between high (Z ≥ 1) *VIM* expression and OS did not reach statistical significance (HR: 1.42 95% CI 0.85–2.38; p = 0.178, Additional file 1: Table S3) in multivariable analysis. However, multivariate analyses showed a significant association between higher *VIM* expression (Z ≥ 2) and shorter overall survival (HR: 3.99 95% CI 1.65–9.66; p = 0.002) (Additional file 1: Table S4).

**Table 3:** Multivariate analysis of disease-free survival of patients with AML for *VIM* expression Z-score ≥ 1 (N = 166)

Variables	Hazard ratio	95% CI		p value
*ln*WBC	1.05	0.87	1.27	0.633
PB blast	1.01	1.001	1.02	0.028
Cytogenetic abnormality	1.82	0.83	4.00	0.134
Cytogenetic risk				
Intermediate	4.76	1.73	13.1	0.002
Poor	3.85	1.49	9.90	0.005
Transplant status	0.98	0.58	1.65	0.926
*FLT3*	2.03	1.21	3.40	0.007
*VIM*	2.42	1.26	4.66	0.008

After age-stratification, high *VIM* expression (Z ≥ 1) was significantly associated with worse overall survival even after adjustment for other risk factors in older patients (HR: 1.99 95% CI 1.04–3.81; p = 0.038, Table 4) but not younger patients (Additional file 1: Table S5). Similarly, higher *VIM* expression (Z ≥ 2) was significantly associated with worse overall survival in older patients (HR: 4.27 95% CI 1.62–11.3; p = 0.003, Additional file 1: Table S6). Additionally, when we exclude patients with t15:17 inversions, higher *VIM* expression (Z ≥ 2) remained significantly associated with worse overall survival (HR: 4.30 95% CI 1.60–11.6; p = 0.004).

**Table 4:** Multivariate analysis of overall survival of AML patients associated with *VIM* expression Z-score ≥ 1 in older patients (age ≥ 60; n = 80)

Variables	Hazard ratio	95% CI		p value
Age	1.02	0.97	1.06	0.451
Cytogenetic risk				
Intermediate	0.90	0.35	2.32	0.828
Poor	3.60	1.18	11.0	0.024
Transplant status	0.274	0.12	0.62	0.002
*DNMT3A*	1.77	0.94	3.32	0.077
*RUNX1*	1.67	0.72	3.86	0.230
*TP53*	1.31	0.57	3.01	0.517
*VIM*	1.99	1.04	3.81	0.038

### *VIM* is hypomethylated in patients with high *VIM* expression

**Fig. 7:**
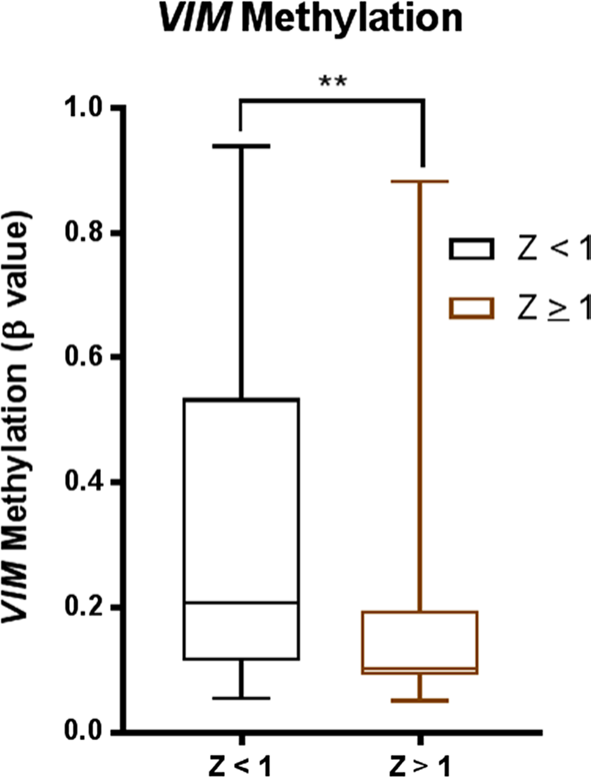
Associations between methylation and *VIM* expression. *VIM* methylation β value comparison between patients according to *VIM* expression (Z-score ≥ 1 and < 1). A non-parametric Mann–Whitney U test was used to compare the median of methylation β value between the groups (p value: 0.0052)

Because *VIM* upregulation was not consistently associated with any particular mutation found in AML, we speculated that other mechanisms might be involved in regulating this gene. In order to examine whether epigenetic changes might play a role in regulating *VIM* expression, we compared methylation β values between *VIM* high (Z ≥ 1) and *VIM* low (Z < 1). We found that *VIM* methylation was significantly lower in the high *VIM* patients compared to that in the low *VIM* patients (Fig. 7; median methylation β value: 0.1 vs 0.2, p = 0.0052).

## Discussion

The epithelial to mesenchymal transition (EMT) is an embryonic developmental process in which epithelial cells transition to cells with a mesenchymal phenotype defined by the loss of E-cadherin and gain of vimentin expression markers [18]. EMT has been shown to play oncogenic roles in cancer progression starting from the initiation of the primary tumor thorough metastasis and resistance to cancer treatment [18]. EMT has also been linked to cancer stem cell survival and maintenance. Importantly, the role of EMT in cancer is not limited to cancer of epithelial origin, but also extend to non-epithelial cancers such as glioma and hematological malignancies [19]. In hematological malignancies, several studies have reported on the role of the EMT transcription factors TWIST1 and TWIST2 in both myeloid and lymphoblastic leukemia [19]. Using an inducible MLL-AF9-driven AML mouse model, a recent study demonstrated that the expression of MLL-AF9 in hematopoietic stem cells (HSC) drives an invasive and chemoresistant AML expressing EMT markers (such as *VIM* among others) [20].

*VIM* is a cytoskeletal protein which overexpression is known to be associated with aggressive disease and worse outcomes in solid cancers such breast, gastrointestinal, and prostate cancers [9–12]. *VIM* regulates EMT, in part, due to its role in regulating cell migration, cell adhesion, EMT signaling pathways and cytoskeletal reorganization [4]. In this study, we characterized the EMT marker *VIM,* in the context of AML. As leukemic cells do not undergo EMT, the molecular functions of *VIM* are less clear in AML. Here we demonstrated that upregulation of *VIM* is associated with poor overall and disease-free survival in older patients with CN-AML. The clinical outcome of AML in older patients who are unable to receive intensive chemotherapy without unacceptable side effects remains dismal. Less than 15% of patients older than 60 years of age survive for longer than 5 years; and the median survival of these patients is only 5–10 months [1]. Whether vimentin protein levels correlate with its mRNA levels and similarly with clinical outcome remains to be examined. The lack of association between high *VIM* and patient’s mutational status is particularly interesting, suggesting other mechanisms such as epigenetic modification may be involved in regulating *VIM* expression. Indeed, we found that patients with high *VIM* (Z ≥ 1) have significantly lower *VIM* methylation in comparison with low *VIM* expression. Consistent with our observation, treatment with 5-Aza-Deoxycytidine, a methylation inhibitor, resulted in several folds increase of vimentin mRNA expression in different colon cancer cell lines [21]. Vimentin expression was also shown to be transactivated by β-catenin/TCF, binding to a site upstream of vimentin promoter in breast cancer cells [22]. Whether similar mechanisms are present in AML is unknown, however the role of β-catenin signaling pathway in maintaining leukemia stem cell survival is well established in AML [23, 24].

## Conclusion

In conclusion, our results demonstrate that *VIM* upregulation is associated with poor clinical outcome in AML. The characterization of *VIM* mRNA expression reveals it as valuable prognostic marker in AML and provides the rationale for further functional and mechanistic investigation into the role of *VIM* in this disease.

## Additional file

**Additional file 1: Figure S1.** *VIM* mRNA expression in sorted AML cells according to their leukemia stem cell markers expression. *VIM* gene expression data obtained from the GSE30377 dataset, in which leukemia blasts obtained from patients with AML (n = 23) were sorted into CD34+CD38−, CD34+CD38+, CD34−CD38−, and CD34−CD38+ populations, *VIM* mRNA levels were compared between the different sorted cell population and unsorted cells. *P < 0.05. **Figure S2.** Survival analysis of AML patients associated with *VIM* expression after stratification of transplant status. (**A**) Overall survival of AML patients with *VIM* expression *VIM* Z-score ≥ 1 and *VIM* Z-score < 1 in patients who did not receive transplant. (**B**) Overall survival of AML patients with *VIM* expression *VIM* Z-score ≥ 1 and *VIM* Z-score < 1 in patients who received transplant. **Table S1.** Clinical Characteristics of 173 AML Patients According to *VIM* Expression Z-Score ≥ 2. **Table S2.** Expression of *VIM* (Z-Score ≥ 2) according to the top mutations present in AML (N = 173 patients). **Table S3.** Multivariate Analysis of Overall Survival of AML Patients Associated with *VIM* Expression Z-Score ≥ 1 (n = 169). **Table S4.** Multivariate Analysis of Overall Survival of AML Patients Associated with *VIM* Expression Z-Score > 2 (n = 169). **Table S5.** Multivariate Analysis of Overall Survival of AML Patients Associated with *VIM* Expression Z-Score ≥ 1 in young patients (Age < 60; n = 89). **Table S6.** Multivariate Analysis of Overall Survival of AML Patients Associated with *VIM* Expression Z-Score ≥ 2 in old patients (Age ≥ 60; n = 80).
