# Supplementary material for: Upregulation of the EMT marker vimentin is associated with poor clinical outcome in acute myeloid leukemia
Source: J Transl Med. 2018 Jun 20;16:170. doi: 10.1186/s12967-018-1539-y (PMC6009962; doi:10.1186/s12967-018-1539-y)
Supplement: Supplementary file 1 — Additional file 1: Figure S1. VIM mRNA expression in sorted AML cells according to their leukemia stem cell markers expression. VIM gene expression data obtained from the GSE30377 dataset, in which leukemia blasts obtained from patients with AML (n = 23) were sorted into CD34+CD38−, CD34+CD38+, CD34−CD38−, and CD34−CD38+ populations, VIM mRNA levels were compared between the different sorted cell population and unsorted cells. *P < 0.05. Figure S2. Survival analysis of AML patients associated with VIM expression after stratification of transplant status. (A) Overall survival of AML patients with VIM expression VIM Z-score ≥ 1 and VIM Z-score < 1 in patients who did not receive transplant. (B) Overall survival of AML patients with VIM expression VIM Z-score ≥ 1 and VIM Z-score < 1 in patients who received transplant. Table S1. Clinical Characteristics of 173 AML Patients According to VIM Expression Z-Score ≥ 2. Table S2. Expression of VIM (Z-Score ≥ 2) according to the top mutations present in AML (N = 173 patients). Table S3. Multivariate Analysis of Overall Survival of AML Patients Associated with VIM Expression Z-Score ≥ 1 (n = 169). Table S4. Multivariate Analysis of Overall Survival of AML Patients Associated with VIM Expression Z-Score > 2 (n = 169). Table S5. Multivariate Analysis of Overall Survival of AML Patients Associated with VIM Expression Z-Score ≥ 1 in young patients (Age < 60; n = 89). Table S6. Multivariate Analysis of Overall Survival of AML Patients Associated with VIM Expression Z-Score ≥ 2 in old patients (Age ≥ 60; n = 80). [file 12967_2018_1539_MOESM1_ESM.docx]

**Additional Figures and Tables:**

**Additional Figures:**

**Figure S1:** *VIM* mRNA expression in sorted AML cells according to their leukemia stem cell markers expression. *VIM* gene expression data obtained from the GSE30377 dataset, in which leukemia blasts obtained from AML patients (n=23) were sorted into CD34+CD38-, CD34+CD38+, CD34-CD38-, and CD34-CD38+ populations, *VIM* mRNA levels compared between the different sorted cell population and unsorted cells. * P<0.05.

**A**

**B**

**Figure S2:** Survival analysis of AML patients associated with *VIM* expression after stratification of transplant status. (**A**) Overall survival of AML patients with *VIM* expression *VIM* Z-score>1 and *VIM* Z-score<1 in patients who did not receive transplant. (**B**) Overall survival of AML patients with *VIM* expression *VIM* Z-score>1 and *VIM* Z-score<1 in patients who received transplant

**Additional Tables:**

**Table S1:** Clinical Characteristics of 173 AML Patients According to *VIM* Expression Z-Score>2

| **Characteristic** | **Z-Score (<2) (n=165)** | **Z-Score (>2) (n=8)** | **p-value** |
| --- | --- | --- | --- |
| **Age, median (years)** | 57 | 69 | 0.036 |
| Young (<60 years) | 90 (54.55%) | 1 (12.5%) | 0.028 |
| Old (>60 years) | 75 (45.45%) | 7 (87.5%) |  |
| **Sex** | | | >0.999 |
| Female (n, %) | 77 (46.67%) | 4 (50.00%) |  |
| Male (n, %) | 88 (53.33%) | 4 (50.00%) |  |
| **FAB** | | | |
| M0 (n, %) | 16 (9.70%) | 0 (0.00%) | >0.999 |
| M1 (n, %) | 42(25.45%) | 2 (25.00%) | >0.999 |
| M2 (n, %) | 36 (21.82%) | 2 (25.00%) | >0.999 |
| M3 (n, %) | 14 (8.48%) | 2 (25.00%) | 0.165 |
| M4 (n, %) | 32 (19.39%) | 2 (25.00%) | 0.659 |
| M5 (n, %) | 18 (10.91%) | 0 (0.00%) | >0.999 |
| M6 (n, %) | 2 (1.21%) | 0 (0.00%) | >0.999 |
| M7 (n, %) | 3 (1.81%) | 0 (0.00%) | >0.999 |
| **WB Count, median** | 15.1 | 69.2 | 0.016 |
| ln (WB Count), mean | 2.591 | 4.0292 | 0.012 |
| **% BM Blast, median** | 72 | 85.5 | 0.061 |
| **% PB Blast, median** | 33 | 65 | 0.022 |
| **Risk Status** | | | |
| Poor (n, %) | 44 (26.7%) | 1 (12.5%) | 0.677 |
| Intermediate (n, %) | 88 (53.3%) | 4 (50.0%) | >0.999 |
| Good (n, %) | 31 (18.9%) | 2 (25.00%) | 0.622 |
| **Cytogenetic Status** | | | 0.256 |
| Normal (n, %) | 75 (45.45%) | 5 (62.50%) |  |
| Abnormal (n, %) | 88 (53.33%) | 2 (25.00%) |  |
| **Transplant Status** | | | 0.141 |
| No (n, %) | 93 (56.36%) | 7 (87.5%) |  |
| Yes (n, %) | 72 (43.64%) | 1 (12.5%) |  |

**Table S2:** Expression of *VIM* (Z-Score >2) according to the top mutations present in AML (N=173 patients)

| **Genes** | **Z-Score (<2) (n=165)** | **Z-Score (>2) (n=8)** | ***p-value*** |
| --- | --- | --- | --- |
| **FLT3 (n, %)** | 47 (28.5%) | 2 (25.0%) | >0.999 |
| **TP53 (n, %)** | 14 (8.48%) | 0 (0.00%) | >0.999 |
| **NPM1 (n, %)** | 45 (27.3%) | 3 (37.5%) | 0.687 |
| **NRAS (n, %)** | 12 (7.27%) | 0 (0.00%) | >0.999 |
| **TET2 (n, %)** | 15 (9.09%) | 0 (0.00%) | >0.999 |
| **RUNX1 (n, %)** | 15 (9.09%) | 0 (0.00%) | >0.999 |
| **CEBPA (n, %)** | 13 (7.88%) | 0 (0.00%) | >0.999 |
| **WT1 (n, %)** | 10 (6.06%) | 0 (0.00%) | >0.999 |
| **DNMT3A (n, %)** | 41 (24.8%) | 1 (12.5%) | 0.681 |
| **IDH1 (n, %)** | 15 (9.09%) | 1 (12.5%) | 0.548 |
| **IDH2 (n, %)** | 15 (9.09%) | 2 (25.0%) | 0.179 |

**Table S3:** Multivariate Analysis of Overall Survival of AML Patients Associated with *VIM* Expression Z-Score>1 (n=169)

| **Variables** | **Hazard Ratio** | **95% CI** | | **p-value** |
| --- | --- | --- | --- | --- |
| Age | 1.02 | 0.99 | 1.03 | 0.054 |
| Cytogenetic Risk | | | | |
| Intermediate | 2.73 | 1.35 | 5.52 | 0.005 |
| Poor | 5.66 | 2.46 | 13.0 | <0.001 |
| Transplant Status | 0.43 | 0.27 | 0.71 | 0.001 |
| *DNMT3A* | 1.54 | 0.97 | 2.45 | 0.067 |
| *RUNX1* | 2.04 | 1.05 | 3.98 | 0.035 |
| *TP53* | 2.13 | 1.04 | 4.38 | 0.039 |
| *VIM* | 1.42 | 0.85 | 2.38 | 0.178 |

**Table S4:** Multivariate Analysis of Overall Survival of AML Patients Associated with *VIM* Expression Z-Score>2 (n=169)

| **Variables** | **Hazard Ratio** | **95% CI** | | **p-value** |
| --- | --- | --- | --- | --- |
| Age | 1.01 | 1.00 | 1.03 | 0.110 |
| Cytogenetic Risk | | | | |
| *Intermediate* | 2.91 | 1.44 | 5.88 | 0.003 |
| *Poor* | 6.13 | 2.65 | 14.2 | <0.001 |
| Transplant Status | 0.40 | 0.25 | 0.65 | <0.001 |
| *DNMT3A* | 1.55 | 0.97 | 2.46 | 0.066 |
| *RUNX1* | 2.25 | 1.15 | 4.40 | 0.018 |
| *TP53* | 2.20 | 1.07 | 4.51 | 0.031 |
| *VIM* | 3.99 | 1.65 | 9.66 | 0.002 |

**Table S5:** Multivariate Analysis of Overall Survival of AML Patients Associated with *VIM* Expression Z-Score>1 in young patients (Age < 60; n=89)

| **Variables** | **Hazard Ratio** | **95% CI** | | **p-value** |
| --- | --- | --- | --- | --- |
| Age | 0.99 | 0.967 | 1.02 | 0.690 |
| Cytogenetic Risk | | | | |
| Intermediate | 3.57 | 1.22 | 10.4 | 0.020 |
| Poor | 4.72 | 1.31 | 17.0 | 0.017 |
| Transplant Status | 0.73 | 0.34 | 1.58 | 0.419 |
| *DNMT3A* | 1.99 | 0.93 | 4.30 | 0.078 |
| *RUNX1* | 5.32 | 1.66 | 17.1 | 0.005 |
| *TP53* | 5.37 | 0.62 | 46.5 | 0.127 |
| *VIM* | 1.02 | 0.38 | 2.72 | 0.969 |

**Table S6:** Multivariate Analysis of Overall Survival of AML Patients Associated with *VIM* Expression Z-Score>2 in old patients (Age > 60; n=80)

| **Variables** | **Hazard Ratio** | **95% CI** | | **p-value** |
| --- | --- | --- | --- | --- |
| Age | 1.01 | 0.96 | 1.05 | 0.727 |
| Cytogenetic Risk | | | | |
| *Intermediate* | 1.01 | 0.40 | 2.58 | 0.976 |
| *Poor* | 4.05 | 1.31 | 12.5 | 0.015 |
| Transplant Status | 0.23 | 0.10 | 0.53 | <0.001 |
| *DNMT3A* | 1.76 | 0.93 | 3.30 | 0.081 |
| *RUNX1* | 1.99 | 0.85 | 4.67 | 0.114 |
| *TP53* | 1.31 | 0.57 | 2.99 | 0.521 |
| *VIM* | 4.27 | 1.62 | 11.3 | 0.003 |
